# Evasion of toll-like receptor recognition by *Escherichia coli* is mediated via population level regulation of flagellin production

**DOI:** 10.3389/fmicb.2023.1093922

**Published:** 2023-03-23

**Authors:** Aaron Tan, Qusai Alsenani, Marcello Lanz, Christopher Birchall, Lauren K. L. Drage, David Picton, Catherine Mowbray, Ased Ali, Christopher Harding, Robert S. Pickard, Judith Hall, Phillip D. Aldridge

**Affiliations:** ^1^Biosciences Institute, Newcastle University, Newcastle upon Tyne, United Kingdom; ^2^Translational and Clinical Research Institute, Newcastle University, Newcastle upon Tyne, United Kingdom; ^3^Urology Department, Freeman Hospital, Newcastle upon Tyne Hospitals NHS Foundation Trust, Newcastle upon Tyne, United Kingdom

**Keywords:** TLR5, UPEC, flagellin, urinary tract infection, bacterial motility

## Abstract

Uropathogenic *Escherichia coli* is a major cause of urinary tract infections. Analysis of the innate immune response in immortalized urothelial cells suggests that the bacterial flagellar subunit, flagellin, is key in inducing host defenses. A panel of 48 clinical uro-associated *E*. *coli* isolates recovered from either cystitis, pyelonephritis asymptomatic bacteriuria (ABU) or UTI-associated bacteraemia infections were characterized for motility and their ability to induce an innate response in urothelial cells stably transfected with a NF-κB luciferase reporter. Thirty-two isolates (67%) were identified as motile with strains recovered from cystitis patients exhibiting an uneven motility distribution pattern; seven of the cystitis isolates were associated with a  > 5-fold increase in NF-κB signaling. To explore whether the NF-κB signaling response reflected antigenic variation, flagellin was purified from 14 different isolates. Purified flagellin filaments generated comparable NF-κB signaling responses, irrespective of either the source of the isolate or H-serotype. These data argued against any variability between isolates being related to flagellin itself. Investigations also argued that neither TLR4 dependent recognition of bacterial lipopolysaccharide nor growth fitness of the isolates played key roles in leading to the variable host response. To determine the roles, if any, of flagellar abundance in inducing these variable responses, flagellar hook numbers of a range of cystitis and ABU isolates were quantified. Images suggested that up to 60% of the isolate population exhibited flagella with the numbers averaging between 1 and 2 flagella per bacterial cell. These data suggest that selective pressures exist in the urinary tract that allow uro-associated *E*. *coli* strains to maintain motility, but exploit population heterogeneity, which together function to prevent host TLR5 recognition and bacterial killing.

## Introduction

Urinary tract infections (UTIs) are among the most common bacterial infections suffered by individuals of all ages. They affect an estimated 150 million people worldwide including children, young adults and older populations (Foxman, 2002). Infections are often painful and debilitating, associated with a wide range of pathogens, but the majority (70–80%) link to the bacterial uropathogen *Escherichia coli* (Klein and Hultgren, 2020). Regardless of the uropathogen, treatment options remain limited with antibiotics being the first choice therapeutic. Treatment consequences, namely multi-drug resistant bacteria, often underpin persistent or rUTIs and have driven the urologic community to work collaboratively to adopt antibiotic stewardship programs (Fisher et al., 2018).

Research to date suggests UTIs link to genotypic and phenotypic variation in both the host and the uropathogen (Hawn et al., 2009; Schreiber et al., 2017). At present it is assumed that the relationship between an individual’s susceptibility and bacterial virulence determines the balance between tolerance of invading pathogens and the mounting of an immune response, which in turn dictates the course of infection and subsequent recurrence (Chan et al., 2013; Choi et al., 2016; Drage et al., 2019). *E*. *coli* reside naturally in the gastrointestinal tract, but are able to migrate from the anus, colonize the vaginal and periurethral areas, then ascend to the bladder causing asymptomatic bacteriuria (ABU) or acute cystitis (Schreiber et al., 2017). However, our understanding of the associated host–microbe interactions is compounded by the observation that the same or related strains can lead to both symptomatic UTI and ABU. One outcome is that ABU patients, particularly the elderly, are often given antibiotics without justification due to clinical uncertainty (Foxman, 2002).

While *E*. *coli* harbors a large array of virulence determinants, the ability to cause UTI is dependent on the ability of the bacterium to ascend the urinary tract through adherence (fimbriae driven) and flagella-based motility (Klein and Hultgren, 2020). Moreover *in vivo* studies using genetically engineered *E*. *coli* strains and mice UTI models support flagella as being a key factor in the aetiology of an UTI (Lane et al., 2005; Wright et al., 2005; Lane et al., 2007). *E*. *coli* is known to produce 2–8 flagella per cell arranged peritrichously across the cell surface and, is characterized genetically, by approximately 60 flagellar genes organized into three loci: *flg*, *flh* and *fli* that function to orchestrate flagellar assembly and rotation (Chilcott and Hughes, 2000; Aldridge and Hughes, 2001). Evidence supports flagellar assembly and function to be coupled to flagellar gene expression by a complex transcriptional hierarchy (Chilcott and Hughes, 2000). Tight control of flagellar gene expression enables *E*. *coli* to efficiently pass through ON/OFF phases of motility that can be exploited and used advantageously during host–microbe interactions (Sim et al., 2017). In fact, motility is a well-recognized pathogenicity, virulence and/or colonization factor for a wide range of bacterial species including uropathogenic *E*. *coli* (Josenhans and Suerbaum, 2002).

Flagellin is the bacterial ligand for the TLR5 receptor and in humans motile uropathogens such as *E*. *coli* are sensed *via* urothelial TLR5 receptors (Andersen-Nissen et al., 2007). TLR5 activation results in the rapid release of urothelial host defence agents including cytokines and defensins that function individually and/or collectively to kill potential uropathogens (Ali et al., 2009; Sivick and Mobley, 2010). However, using urine and employing *in vitro* chemotaxis assays Herrmann and Burman (1985) reported that only 68% (19/28) of *E*. *coli* isolates associated with cystitis were motile. Yet, there is strong evidence to support *E*. *coli* exploiting flagellar-mediated movement to establish the initial ascending colonization of the bladder from the urethra (Schaeffer et al., 1987; Wright et al., 2005). Lane et al. (2005) and Wright et al. (2005) both concluded that motility provided *E*. *coli* a competitive advantage over non-motile *E*. *coli* strains in establishing an UTI in murine models. A key challenge therefore is to understand how uropathogenic bacteria regulate their motility in the urinary tract.

Using clinically derived uro-associated *E*. *coli* isolates data are presented suggesting a regulatory mechanism linked to population heterogeneity that maintains motility within a bacterial population, but at levels below a threshold required for innate immune recognition.

## Results

### Uro-associated *E. coli* motility and the urothelial innate response

**Figure 1:**
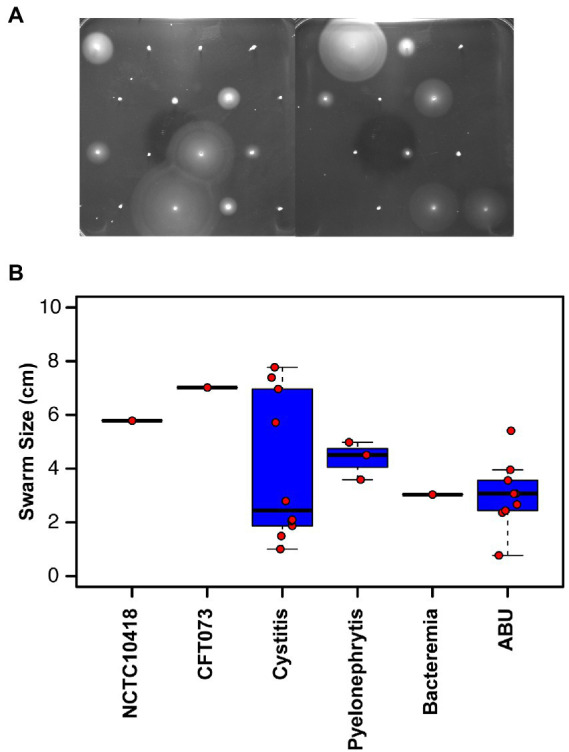
Motility of clinical uro-associated isolates. **(A)** Clinical *E*. *coli* isolates at 8 h in motility agar assay data showing the diverse range of phenotypes. Strains shown are in numerical order starting from 3,398 to 3,425 (Supplementary Tables S1, S2). **(B)** Quantification of swarm diameter for *n* = 3 independent colonies of each clinical isolate. Individual values and the members of each subset are declared in Supplementary Table S1.

Uro-associated *E*. *coli* isolates (Total: 48) were curated between 2010 and 2015 from patients presenting with either cystitis, pyelonephritis, asymptomatic bacteriuria (ABU) or UTI-associated bacteraemia. Two reference *E*. *coli* strains, CFT073 (Welch et al., 2002) and NCTC10418 (Ali et al., 2017) were used as controls. The motility phenotypes of the isolates were assessed using semi-quantitative agar assays (Figure 1A) and 32/48 (67%) of the clinical isolates were identified as motile (Supplementary Table S1). In general, the swarms of isolates recovered from ABU patients measured between 0.8 and 5.4 cm, while cystitis strains exhibited an uneven motility distribution pattern with strains swarming less (*n* = 6) or greater (*n* = 4) than 5.4 cm, respectively (Figure 1B; ANOVA *p* < 0.005).

**Figure 2:**
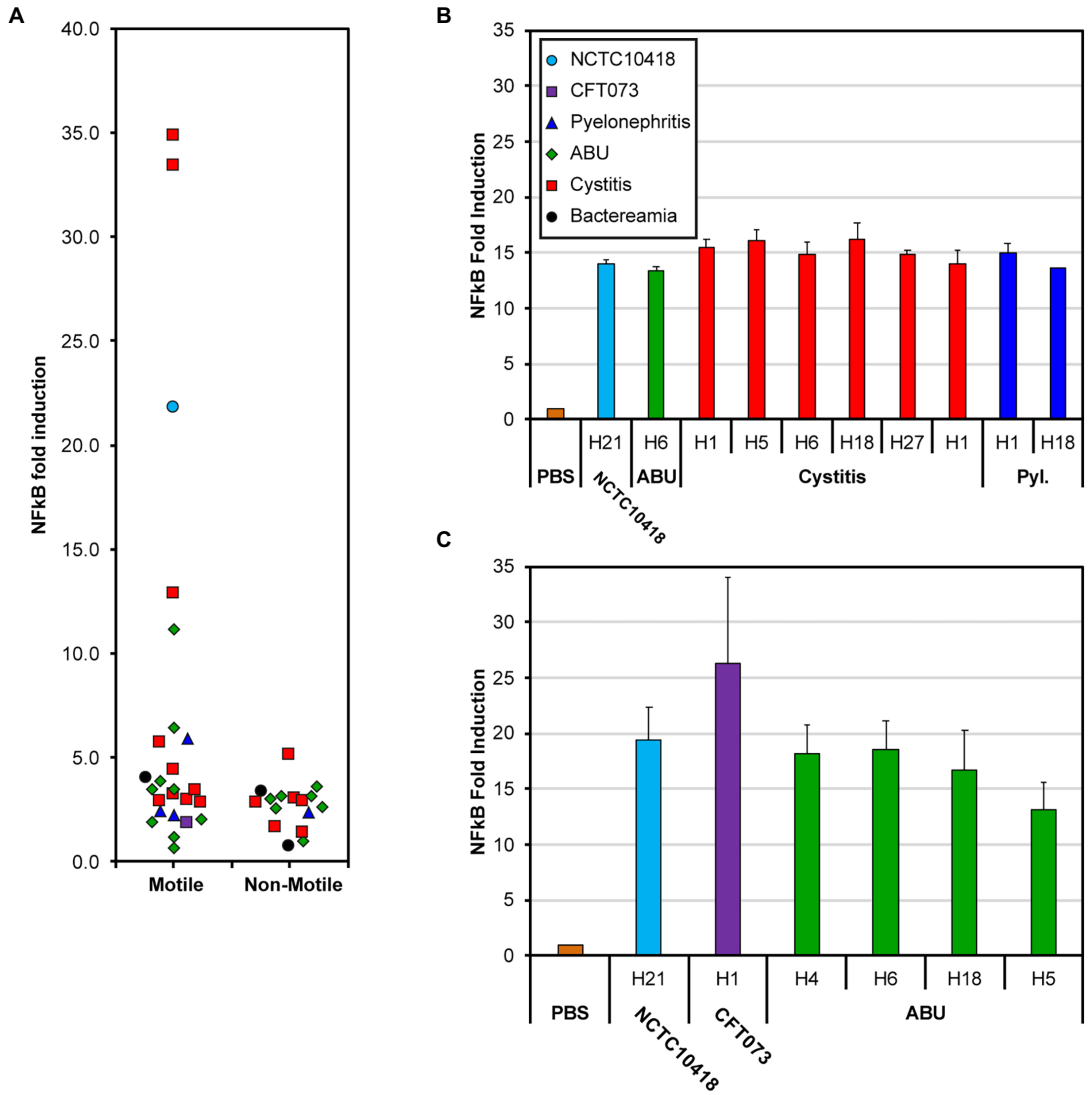
NF-kB response of bladder RT4 cells challenged with whole cells and flagellin. **(A)** NF-κB fold induction of motile and non-motile *E*. *coli* clinical isolates [Supplementary Table S1 (3,398–3,710) and Supplementary Table S2 (3,399–3,707)]. Points have been scattered left or right with respect to the x-axis for clarity. Colors and point shape as shown in the key of panel **(B)** represent the associated type of infection of the isolate **(B)** NF-κB response of RT4 bladder cells challenged for 24 h with 250 ng/mL flagellin filaments isolated from 11 different *E*. *coli* strains; from left to right: NCTC10418, 3,419, 3,408, 3,409, 3,411, 3,412, 3,414, 3,417, 3,398, 3,424 (Supplementary Table S1). **(C)** NF-κB fold increase of RT4 bladder cells challenged with 50 ng/ml flagellin filaments isolated from 6 different *E*. *coli* strains; from left to right: NCTC10418, CFT073, 3,692, 3,693, 3,694, 3,698 (Supplementary Table S1). Strain choice in **(B,C)** reflects the diversity in whole cell challenge responses presented in **(A)**. Error bars represent the standard deviation. All data is an average of a minimum of *n* = 3 independent biological repeats with *n* = 2 technical repeats for NF-κB assays.

The impact of bacterial motility on the urothelial innate response was assessed *in vitro* using 24-h challenges of heat-killed isolates (1 × 10^5^ CFU/ml) and bladder RT4 cells stably transfected with a NF-κB luciferase reporter (Ali et al., 2017). Following these challenges 33/42 (78%) of the clinical isolates and the control strain CFT073 were associated with a 2–5 fold increase in NF-κB signaling activity compared to a PBS control (Figure 2A; *p* = 0.029). Seven motile isolates (Supplementary Table S1: 3406, 3,408, 3,411, 3,412, 3,414, 3,419, 3,424) equating to 30% of all motile strains and predominantly associated with cystitis, and the control strain NCTC10418 exhibited increases of >5-fold. Two of these isolates, 3,411 and 3,414, generated NF-κB signaling activities of >30 fold (Figure 2A) and swarm sizes of >7 cm (Supplementary Table S1).

### Urothelial responses to flagellins prepared from uro-associated *E. coli* isolates

UTIs are ascending infections and urothelial cells respond to potential uropathogens *via* flagellin detection, TLR5 signaling and the release of antimicrobial killing and proinflammatory agents (Ali et al., 2017). TLR5 proteins recognize a conserved motif found in the majority of flagellins (Andersen-Nissen et al., 2005) while antigenic variation amongst flagellins has defined 53 flagellin or H-serotypes in *E*. *coli* (Wang et al., 2003). Characterization of the clinical isolates used in these analyses identified seven H-serotypes, H1, H4, H5, H6, H18, H27 and H21 (Supplementary Table S1). To explore whether the signaling response reflected antigenic variation, flagellin was purified, in the form of flagellar filaments, from the control strains and 13 of the *E*. *coli* isolates associated with either ABU, cystitis or pyelonephritis infections (Supplementary Table S1), and the urothelial cell challenges repeated (Figures 2B,C). Compared to the PBS control, purified flagellin filaments from the 15 different preparations generated robust and comparable NF-κB signaling responses when used at concentrations of either 250 or 50 ng/ml (Figure 2B; ANOVA *p* = 0.13; Figure 2C; ANOVA *p* = 0.12). These measured responses did not reflect either the source of the isolate or H-serotype and argued against host variability, between isolates, being related to flagellin itself.

### Growth characteristics of strains

**Figure 3:**
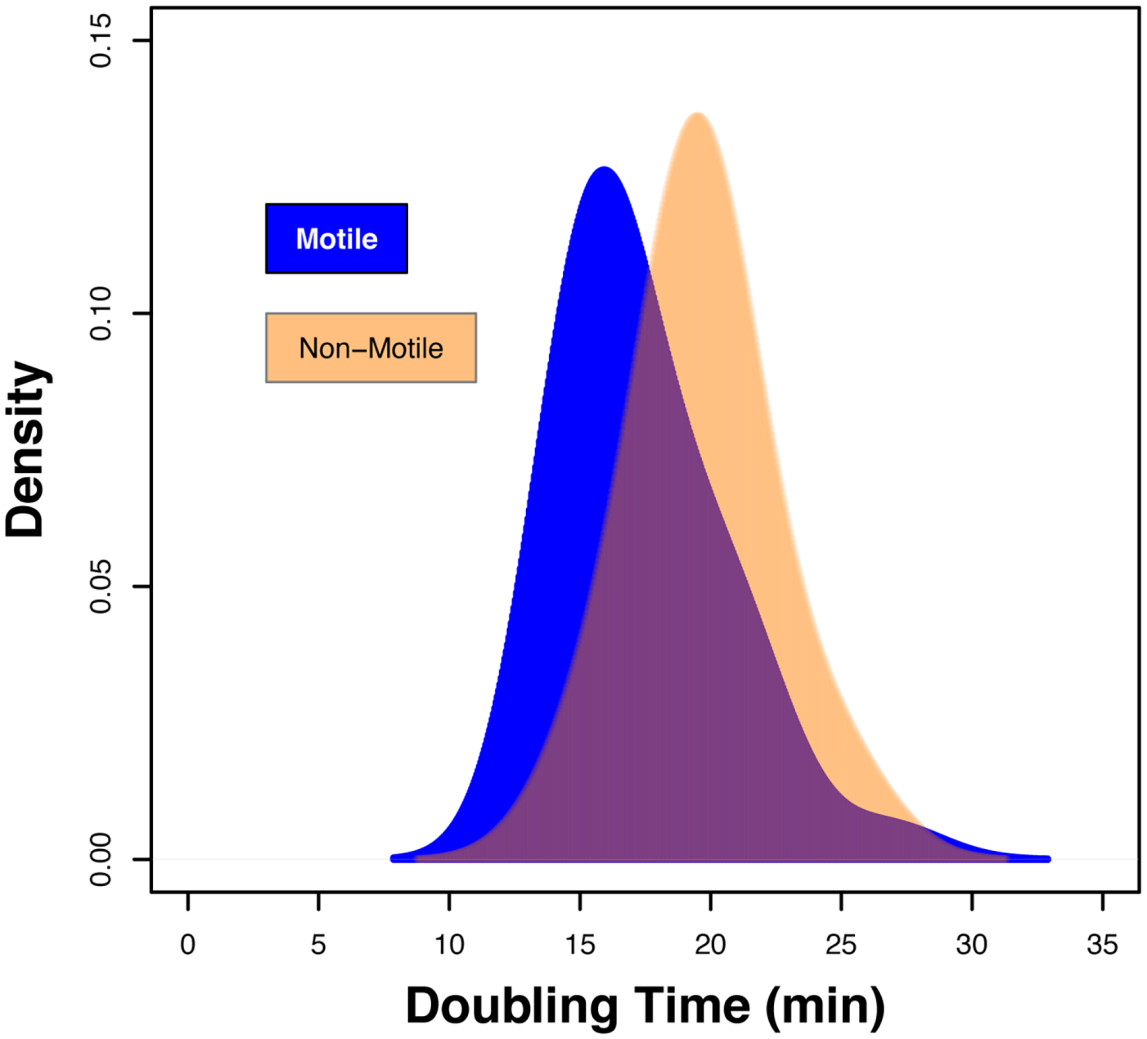
Fitness of isolates defined by the maximum doubling time (min). Density plot showing the range of doubling times for motile clinical strains 3,398 to 4,745 declared in Supplementary Table S1 and non-motile isolates 3,399 to 3,707 in Supplementary Table S2. Data presented is based on *n* = 3 independent biological repeats.

Using chemostat growth conditions the growth rate of *E*. *coli* strain RP437 was shown to correlate with flagellar abundance, suggesting that a change in growth fitness could impact host recognition (Sim et al., 2017). To explore whether the differences in host response linked to fitness, the maximum doubling times of the clinical isolates and the two control strains were explored. The maximum doubling time of all strains varied between 13.9 and 26.9 min, respectively (Supplementary Tables S1, S2). Density plot analysis, stratified for motility, identified a shift between these two sets of strains (Figure 3). The average maximum doubling time for the motile strains was 17.4 ± 3.0 min compared to 19.7 ± 2.5 min for the non-motile strains (Figure 3; ANOVA *p* = 0.011). However, the range of maximum doubling times within each grouping was similar (Motile: 13.9–26.9 min.; Non-Motile: 14.7–25.3 min.). These data argued against fitness being a primary driving factor in the variation in host recognition observed within the motile subset of the strain collection.

### Urothelial responses to outer membranes prepared from uro-associated *E. coli* isolates

**Figure 4:**
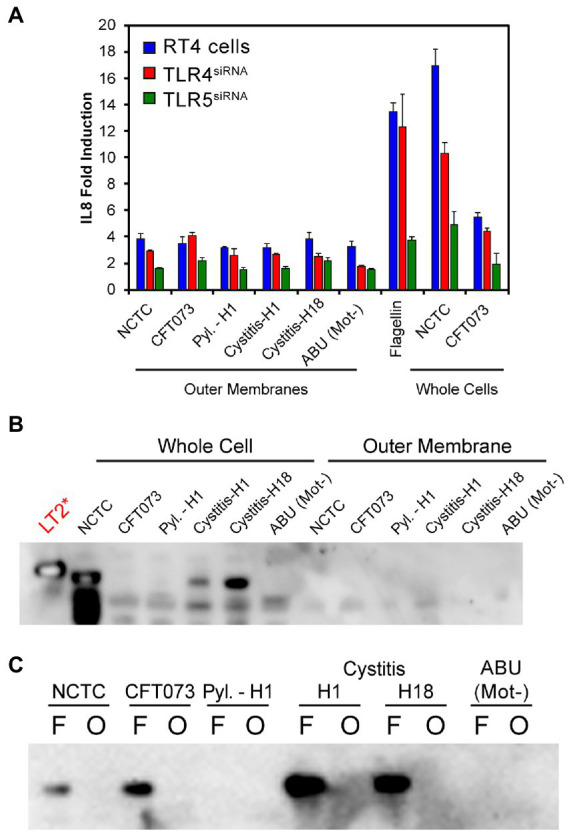
**(A)** IL-8 fold induction compared to a PBS control of RT4 cell media following transfection with siRNAs targeting either TLR4 or TLR5 expression and challenging for 24 h with either outer membrane preparations, flagella filaments (50 ng/mL) or heat killed whole bacteria. Data represents the average of a minimum of *n* = 3 biological repeats and *n* = 2 technical repeats of all assays. **(B)** Immunoblot using α-FlgE_ST_ on whole cell lysates of strains used in panel **(A)** and the outer membrane preparations. *S*. Typhimurium strain LT2 is used as a positive control. No detectable FlgE was observed in the outer membrane preparations. **(C)** Comparison of flagellar filament preparations (F) and outer-membranes (O) of strains used in panel **A**. In most cases FlgE is detectable in the flagellar preparations as expected due to the shearing method used to isolate the filaments. No outer-membrane preparation showed a FlgE band.

While *in vitro* and clinical studies suggest TLR5 to be the key host receptor in detecting and protecting the bladder from uropathogenic *E*. *coli*, murine studies and genotype analyses also support a role for TLR4 (Samuelsson et al., 2004; Andersen-Nissen et al., 2007; Ragnarsdottir et al., 2007; Song et al., 2009; Smith et al., 2011; Ali et al., 2017). TLR4 recognizes lipopolysaccharide, the major outer membrane (OM) component of Gram-negative bacterial species such as *E*. *coli* (Tapping et al., 2000). Potential roles for TLR4 and LPS in the variable recognition of *E*. *coli* were further explored using outer membrane preparations from three motile isolates [3,398 (Pyl.-H1), 3,408 (cystitis-H1) and 3,412 (cystitis-H18)] (Supplementary Table S1), one non-motile isolate [3,416 (ABU (Mot^−^)] (Supplementary Table S2) and the two control strains NCTC10418 and CFT073 (Figure 4). These were used to challenge RT4 (TLR^+^) cells and RT4 cells where either TLR4 or TLR5 expression had been inhibited by siRNA knockdown (Supplementary Figure S1; Mowbray et al., 2018). The innate response was determined through quantification of the pro-inflammatory cytokine IL-8 (Figure 4A). Data indicated that the IL-8 responses to flagellin (50 ng/ml), whole bacterial and outer membrane (OM) sample challenges were significantly reduced following challenge of the TLR5^siRNA^ cells compared to wild-type (TLR^+^) cells (ANOVA *p* < 0.001). Analyses of the IL-8 responses following challenge of the TLR4 ^siRNA^ cells were more strain variable and supported minor roles for LPS, and TLR4 in the RT4 bladder cell innate response to an acute infection.

Effector IL-8 responses (< 2-fold) were observed in the TLR5^siRNA^ cells challenged with OM preparations. These were linked, potentially, to contamination of the preparations with flagellin, even though surface appendages were sheared by blending before OM isolation (see methods). To determine potential flagellar contamination residual levels of the flagellar hook protein FlgE (Paradis et al., 2017), were targeted. This approach addressed any serotype restrictions associated with using antibodies raised against specific flagellins. FlgE from *Salmonella enterica* serovar Typhimurium and *E*. *coli* exhibit 88% identity and 93% similarity (McClelland et al., 2001; Welch et al., 2002), which allowed an antibody raised against *S*. Typhimurium FlgE, α-FlgE_ST_ (Minamino and Namba, 2008), with cross reaction between the species (Figure 4B) to be used to identify potential contamination of the OM preparations. The α-FlgE_ST_ antibody was only able to detect FlgE in either whole cell lysates or flagellar preparations (Figures 4B,C) of motile isolates with no signal detected in the ABU (Mot^−^) samples. These data therefore argued that flagellin is the key factor in activating the bladder innate response to potential *E*. *coli* infections with LPS invoking a minor response.

### Correlating urothelial responses to flagellar abundance amongst uro-associated *E. coli* isolates

**Figure 5:**
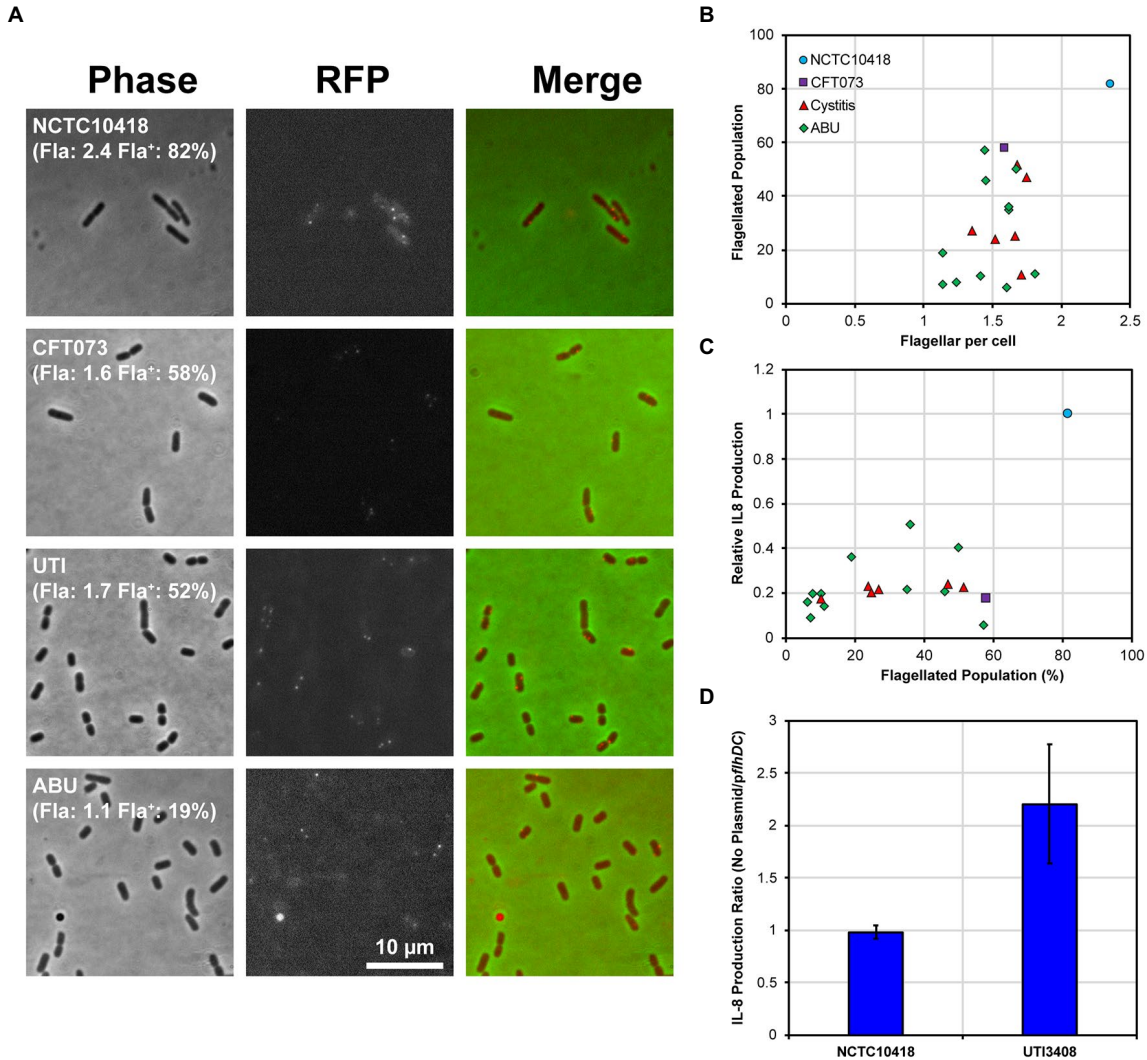
Uro-associated *E*. *coli* population heterogeneity and UPEC evasion of the TLR5 response. **(A)** Example regions of phase contrast and fluorescent images of FlgEA240C foci in the control strains NCTC10418 and CFT073, and two uro-associated clinical isolates. Quantification of a minimum of 250 cells per strain is shown in brackets where Fla: = average number of FlgE foci per cell and Fla^+^: = percentage of the population with foci. Images are chosen to show foci and may not reflect quantified numbers. **(B)** Scatter plot showing average foci per cell versus flagellated population (the percentage of cells having FlgE foci). The range of average foci per cell for all strains except NCTC10418 are within experimental error (*p* = 0.143). Regression analysis identified a 18.4% standard error of regression and an *R*^2^ value of 0.33 and a *p*-value = 0.008, consistent with the observed variation. **(C)** Scatter plot showing percentage of flagellated population versus relative NF-κB induction (control strain NCTC10418 = 1.0). Standard error of regression = 0.17, *R*^2^ value = 0.30, *p*-value = 0.012. **(D)** IL-8 production, presented as a ratio, following challenges of RT4 cells with NCTC10418 (p*flhDC* -ve) or strain 3,408 (p*flhDC* transformed).

The variability in host response, observed in Figure 2A, could not be explained by LPS alone so the roles of flagellar abundance were investigated. This was facilitated by exploiting a flagellar hook gene variant *flgEA240C* that allowed malemide-cysteine crosslinking of a fluorophore to assembled flagellar hooks. Visualization of the hooks by fluorescence microscopy (Sim et al., 2017), then allowed quantification of the hook numbers of cystitis (Schreiber et al., 2017), ABU (Lane et al., 2005) and PYL (Foxman, 2002) isolates (Figure 5). Individual flagella were visualized as distinct foci (Figure 5A) with the numbers of foci counted determining the number of flagella expressed by individual bacterial cells. Analysis of all clinical isolates was prevented by their antibiotic profiles restricting the use of pBAD*flgEA240C*, which uses kanamycin as the selective agent. However, data relating to 6 cystitis and 11 ABU strains (Supplementary Table S1), were collated, which equated to 50% of the motile strains investigated in this study.

The control *E*. *coli* strain NCTC10418 averaged 2.4 foci or flagella per cell, while the clinical isolates and CFT073 averaged between 1 and 2 flagella per cell (ANOVA *p* = 0.148; Figure 5B) x-axis. Data also suggested that only 10 to 60% of the CFT073 and clinical isolate populations exhibited FlgEA240C foci compared to 80% of the NCTC10418 cell population (Figure 5B) y-axis. Comparison of these data to IκBα protein levels during flagellin challenge (Supplementary Figure S2) and urothelial IL-8 responses (Figure 5C; ANOVA *p* = 0.013) strongly suggested that uro-associated *E*. *coli* strains exploit population heterogeneity to preserve motility and avoid detection by TLR5. It is therefore predicted that variability in the host response, i.e., whether symptoms will or will not present, is linked to population heterogeneity and flagellar abundance of the invading uropathogen.

### Driving recognition via gene regulation

Expression of the *E*. *coli* flagellar system is regulated by a transcriptional hierarchy controlled by the master transcriptional regulatory FlhD_4_C_2_ (Wang et al., 2006). FlhD_4_C_2_ levels are sensitive to a wide range of regulatory mechanisms that include transcription, translation and protein stability (Claret and Hughes, 2000; Soutourina and Bertin, 2003; Kitagawa et al., 2011; Takaya et al., 2012). To further examine the link between a population of uro-associated *E*. *coli* and flagellar gene expression, a high copy number plasmid encoding *flhDC* was transformed into the control strain NCTC10418 and isolate 3,408 (Cystitis; 25% Fla^+^; 1.66 Fla^+^/cell; 0.19 relative IL-8 production: Supplementary Table S1) and RT4 urothelial cell challenge experiments performed. IL-8 data, reflecting the host response, showed that increasing *flhDC* expression in 3408 resulted in a two-fold increase in IL-8 concentrations but no change for NCTC10418 (Figure 5D).

## Discussion

*E. coli* swim efficiently with only one flagellum per cell (Mears et al., 2014). The observations that uro-associated *E*. *coli* clinical strains were characterized by 1 to 2 flagella per cell (Figure 5), which supported motility and linked to dampened host innate effector responses, predicted a mechanism by which *E*. *coli* are able to colonize the lower urinary tract environment. Essentially these data suggest that uro-associated bacterial populations adopt a “bet-hedging” strategy, which involves manipulating flagellar production and motility to avoid the host TLR radar systems, which functions to maximize their survival and colonization of the urogenital tract (Veening et al., 2008). However, once bacterial motility is enhanced through increased flagellar production, modelled *in vitro* using strain 3,408 (Figure 5D), microbial detection by urothelial cell TLRs, typically TLR5, is triggered resulting in bacterial killing. These conclusions are based on the analysis performed using a defined, although not trivial, number of uro-associated clinical isolates: 48 in total.

Flagellar systems have been shown to be subject to multiple controls (Brown et al., 2009) and uro-associated *E*. *coli* regulating flagellar synthesis and bacterial motility to ensure its survival is not a unique concept. For example, *S*. *enterica* generates a bipolar Fla.^−^/Fla.^+^ population in response to either nutritional and/or cell envelope stresses (Koirala et al., 2014), while *Caulobacter crescentus* divides asymmetrically to produce one motile cell each division, ensuring a subpopulation of motile progeny within a growing population (Jenal, 2000). The question why *E*. *coli* does not completely switch off flagellin synthesis to evade TLR5 recognition probably links to its ability to survive in urine, a nutritionally weak growth medium (Alteri et al., 2009). However, the concept that immune evasion, i.e., host TLR5 recognition of flagellin proteins drives uro-associated *E*. *coli* to downregulate flagellar production may help unravel the pathogenesis of asymptomatic bacteriuria (ABU), defined as the presence of bacteria in the urinary tract without inflammatory symptoms. This model is consistent with previous findings showing flagellar gene expression is down-regulated in the bladder (Snyder et al., 2004; Lane et al., 2007) but induced when bacteria ascend to the kidneys (Lane et al., 2007). However, our data and proposed model argue that down regulation is not a complete ON/OFF switch allowing for a sub-population of bacterial cells to maintain motility and the capacity to ascend further up the urinary tract.

To establish an UTI it has been argued that infecting bacteria need to divide rapidly to survive the host innate response with bacterial doubling times of between 17 to 34 min reported (Forsyth et al., 2018). Phenotypic data using uro-associated clinical isolates support bacterial motility to be associated with a faster average maximum doubling time (Figure 3). Growth experiments using steady state chemostat cultures have also shown that faster growing *E*. *coli* produce more flagella (Sim et al., 2017). As *E*. *coli* motility is linked to host detection and bacterial killing these data support a model whereby rapid growth results in more flagella, but also higher cell densities, which allow bacteria to overwhelm the host defenses (Sintsova et al., 2019), and present clinically as UTIs.

A potential limitation of this study is that the *E*. *coli* strain panel analyzed was biased and did not reflect the true diversity of UPEC. However, it can be argued that the heterogeneous nature of the 48 strains studied in relation to their motility phenotype, flagellin and LPS serotypes showcased the diverse phenotypes of uropathogenic *E*. *coli* clinical isolates compared to typical laboratory strains, e.g., CFT073. Such heterogeneity also highlighted the need to appreciate and account for such diversity when modelling host–microbe interactions *in vitro*. When assessing host TLR recognition of pathogen associated molecular patterns, such as LPS and flagellin, it is extremely difficult to ensure 100% purity of the isolated bacterial components, particularly flagellin (Acharya et al., 2019). However, the fact that the flagellin/TLR5 signaling response in urothelial cells is increased by 10 fold compared to the LPS/TLR4 response suggests a minimal role for LPS in the bladder host response and justifies the flagellin isolation protocol used in this study (Ali et al., 2017). Similar limitations also applied to ensuring the purity of the outer membrane preparations, so in this case flagellin contamination was excluded using a cross-reacting antibody to the FlgE flagellar subunit that does not exhibit antigenic variation.

A key question relates to the cues in the urogenital tract that trigger bacterial growth and facilitate an *E*. *coli* infection, characterized by host inflammatory agents and defined clinically as a symptomatic UTI episode (Sivick and Mobley, 2010). It is generally accepted that low nutrient conditions up-regulate *E*. *coli* flagellar synthesis *via* activation of the *flhDC* operon (Wada et al., 2012) although other signals including urine osmolality and pH cannot be ignored (Reitzer and Zimmern, 2019). Studies in *Salmonella* grown in low nutrient conditions have shown that non flagellar regulators such as RflP, a regulator that modulates ClpXP recognition of FlhD_4_C_2_, can also impact the activity of the master regulator FlhD_4_C_2_ and hence flagellin synthesis (Takaya et al., 2012; Wada et al., 2012; Spurbeck et al., 2013; Koirala et al., 2014). Whether comparable regulators function to trigger flagellar growth in uro-associated *E*. *coli* is not known although NarL, ModE, Metj, GadE, and YdeO, all sensors of environmental cues, have been identified as playing potential roles in infection-specific uro-associated *E*. *coli* gene expression (Sintsova et al., 2019). This study was not designed to identify the regulatory mechanisms functioning to control flagellin synthesis in the urogenital tract, hence environmental and genetic cues including population densities, urine osmolality and electrolytes, urinary and bacterial metabolites, and pH need to be investigated further.

Population heterogeneity is not an original concept and has been shown to be exploited by a number of bacterial species to retain a selective advantage particularly during growth in specific environmental niches (Veening et al., 2008; Stewart and Cookson, 2012; Casadesús and Low, 2013). However, its exploitation by uro-associated *E*. *coli* to regulate flagellin synthesis and avoid the host defenses is novel. In relation to the pathology of an UTI these data suggest a model in which *E*. *coli* regulate their flagella numbers to survive and colonize the uro-genital tract. ABU (Chan et al., 2013; Drage et al., 2019), is often described as tolerance of the host to *E*. *coli*, but probably reflects a physiological state whereby the flagellin threshold to activate TLR5 signaling has not been reached (Supplementary Figure S3). ABU is particularly common in older patients with weakened innate immune responses (Phillips et al., 2012). This suggests that the flagellin threshold may change with age and potentially is influenced by variable urogenital TLR5 expression levels exhibited by individuals susceptible to recurrent UTI.

## Materials and methods

### Strains and general microbiology

*E*. *coli* strains used in the study are described in Supplementary Tables S1, S2; Strains 3,398 to 3,710 were a kind donation from the Diagnostic Microbiology Unit at the Freeman hospital, Newcastle NHS Trust, Newcastle upon Tyne between 2010 and 2012. No ethics were necessary for the use of these strains as the researchers did not have access to clinical records and the only information provided by the unit was the type of UTI associated with each isolate. Strains 4,738–4,745 came from the clinical study of Drage et al. (2019) that was conducted under an ethically approved study protocol (ref: REC14-NE-0026).

Strains used during this study were propagated in or on Luria-Bertani (LB) medium using 1.5% agar for plates. Incubation, unless stated otherwise, was overnight at 37°C with liquid cultures aerated by orbital shaking at 160 rpm. All motility assays were performed by either direct inoculation using a toothpick or inoculating 3 μl of an overnight culture onto motility agar (1% Tryptone, 0.5% NaCl, 0.3% Agar) and incubating for 8 h at 30°C. Images of motility swarms were digitally captured, and the vertical and horizontal diameter measured to generate an average swarm distance using ImageJ. All swarm assays were performed with a minimum of three independent colonies. Transformation of the plasmids p*flhDC* or pBAD*flgE*A240C were performed by electroporation as described previously (Schaeffer et al., 1987). Selection for plasmids was performed using either 100 μg/ml Ampicillin or 50 μg/ml Kanamycin. p*flhDC* was generated by cloning a PCR product using the primers *flhD*-42FBam [ggcggatccGGGTGCGGCTACGTCGCAC] and *flhC* + 616RBam [ggcggatccCGCTGCTGGAGTGTTTGTCC] into the high copy number vector pSE280 using standard cloning techniques.

The maximum doubling time was determined from growth curves of all strains grown in LB media at 37°C. Strains were grown overnight and diluted to a starting OD_600_ of 0.02. Two hundred microliters were aliquoted into a 96-well plate leaving the outer wells empty. The plate was sealed with a BreatheEasy membrane and OD_600_ measurements taken every 400 s for 10 h in a BMG Fluostar microplate reader, with orbital shaking between measurements. Data was processed in R Studio and coding is available on request. Background was subtracted, and all negative values were set at 0.02 defined as the calculated starting OD_600_. The maximum slope for each strain was derived from log(OD_600_) using a sliding sub-set of 10 time points (~60 min) across the timeline of the growth experiment. The maximum doubling time was derived from the calculated maximum slope before the mean value was determined. All growth experiments were conducted 3 times using independent colonies.

### Isolation of flagellin and outer membranes

All flagellin and outer membrane (OM) preparations were based on 1 l cultures of strains grown to an OD_600_ of 0.6–0.7. Cells were centrifuged at 3,890 *g* and cell pellets resuspended in cold 10 mM HEPES pH 7.4. For flagellin isolation, cell suspensions were sheared using an Ultra-Turrax blending stick for 2 min set at 13,500 rpm. The same protocol was used prior to OM isolation to reduce flagellin contamination. Blended supernatants were centrifuged at 100,000 g for 1 h at 4°C to collect sheared flagellar filaments. The pellets were washed by repeating this procedure three times. Pellets were resuspended in 10 mM HEPES pH 7.4 and centrifuged at 3890 *g* to improve the removal of cell debris between each ultra-centrifuge wash step. The washed flagellin pellets were resuspended in 500 μl 10 mM HEPES pH 7.4 and stored at −20°C.

For outer membrane isolation cell suspensions were lysed using a Constant Systems cell disruptor at 23kPSI. Lysed cell suspensions were centrifuged at 12,000 *g* at 4°C for 40 min, the supernatant layered onto a sucrose gradient and centrifuged at 56,000 *g* for 36 h at 4°C. Outer membrane fractions were resuspended and washed once in 10 mM HEPES pH7.4. The washed outer membrane fraction was collected by centrifuging at 134,000 *g* for 6 h at 4°C and the resulting pellet resuspended in 500 μl 10mmM HEPES. The quality of preparations was assessed using standard SDS polyacrylamide gel electrophoresis.

### Immunoblots

Cell lysate normalized to OD_600_ (Aldridge et al., 2006) and outer membrane samples (0.5 μg) were separated using a NuPAGE™ 4 to 12%, Bis-Tris, 1.0–1.5 mm, Mini Protein Gel. Proteins were transferred onto a nitrocellulose membrane (GE Healthcare) using the Bio-Rad Trans-Blot Turbo Transfer system (at 1.3 A, 25 V for 7 min). Membranes were blocked overnight at 4°C in 5% skimmed milk powder; 0.1% Tween 20 PBS solution (Aldridge et al., 2006). Following washing the membranes were incubated for 1 h with 1:10,000 dilution of α-FlgE_ST_ antibody (Minamino and Namba, 2008), washed and further incubated 1 h with 1:10,000 dilution of horseradish peroxidase (HRP)-conjugated Goat α-Rabbit antibody (Southern Biotech). After washing blots were exposed to Clarity Max Western ECL substrate (Bio-rad, 1:1 ratio) for 5 min and data captured using an iBright™ Imaging System (Invitrogen).

### NF-κB reporter assay, IL-8 ELISA and immunoblots

Growth conditions for the bladder RT4 cell line has been previously described (Ali et al., 2017). All challenges were performed using 24 well plates seeded with 500,000 cells in 500 μL, and the cells grown until 80–90% confluent. Bladder RT4 cells were challenged in triplicate with either isolated outer membrane preparations (100 ng/ml protein content), flagellin (0–250 ng/mL) or heat-killed whole cells at 37°C and 5% CO_2_ (Ali et al., 2017). Heat-killed whole cells of each bacterial strain were prepared as previously described (Ali et al., 2017). Strains were grown to late log phase (OD600 = 0.6–0.8), cells harvested by centrifugation and resuspended in PBS prior to heating for 15 min at 100°C. No further centrifugation steps were conducted prior to the challenges allowing for intact and heat depolymerized flagellin to remain in the suspension. Prior to heating an aliquot was taken to numerate the colony forming units to calculate the necessary volume required to challenge with 1 × 10^5^ CFU/ml. Challenges were stopped after 24 h, the extracellular media collected and stored at −20°C. Interleukin 8 concentrations (pg/ml) were assayed using an eBioscience IL-8 ELISA kit following the manufacturer’s instructions. Measurement of RT4 NF-κB reporter activity was as previously described (Ali et al., 2017). For IκBα immunoblots, challenged RT4 cells were lysed in RIPA Buffer collected, quantified using a Micro BCA protein assay kit (Thermo) and stored at −20°C before 10 μg was used for immunoblots with an α-IκBα antibody (New England Biolabs).

### Inhibition of TLR4 and TLR5 siRNA expression

TLR4 and TLR5 knockdown experiments using RT4 cells and siRNA were performed as described previously (Mowbray et al., 2018). siRNAs used were as follows: s14196 (TLR4) and s14199 (TLR5) and AM4611 (negative siRNA #1) as a control.

### Quantification of flagellar abundance

Expression of *flgEA240C* was analyzed following bacterial growth at 37°C in LB media containing 0.1% arabinose with shaking until an OD600 of 0.6 to 0.7. Staining of the cells was performed using malemide conjugated AlexaFluor 568 (Sim et al., 2017). Bacterial cell suspensions were immobilized on a 1% agarose padded microscope slide and samples analyzed in triplicate at 100x objective using a Nikon Eclipse Ti inverted microscope capturing both phase contrast (100 ms exposure) and red channel images (1,000 ms exposure) at five different fields of view. Five randomly chosen fields were analyzed manually using the ImageJ cell counter plugin generating data where *n* = 200–300 cells. Foci per cell was averaged across the five fields of view to enumerate the level of cell flagellation, as well as the distribution of flagella over the population.

### Data analysis and presentation

Data and statistical analysis were performed using R Studio and MS Excel including the use of ANOVA and regression analysis where appropriate. Images for figure panels were processed and cropped using ImageJ and imported into Adobe illustrator for formatting. All figures were collated in Adobe illustrator to achieve the correct resolution.

## Data availability statement

The raw data supporting the conclusions of this article will be made available by the authors, without undue reservation.

## Author contributions

AT, QA, ML, CB, LD, DP, and CM designed and conducted the experiments. AA, CH, RP, JH, and PA supervised the project. AT, QA, CM, JH, and PA collated the data and assembled the figures. JH and PA wrote the manuscript. All authors edited the drafted manuscript.

## Funding

Funding for this project has included a self-funded Ph.D. thesis aided by a Newcastle University Overseas Research Scholarship for AT, a sponsored studentship from University of Hail, Saudi Arabia for QA, a Newcastle University William Harker Studentship for ML, a BBSRC DTG Ph.D. studentship for CB, and a non-clinical Ph.D. studentship for LD provided by the NIHR Newcastle Biomedical Research Centre awarded to the Newcastle upon Tyne Hospitals NHS Foundation Trust and Newcastle University. The contribution of DP was a self-financed MPhil thesis project. A Wellcome Trust Clinical Training Fellowship funded AA.

## Conflict of interest

The authors declare that the research was conducted in the absence of any commercial or financial relationships that could be construed as a potential conflict of interest.

## Publisher’s note

All claims expressed in this article are solely those of the authors and do not necessarily represent those of their affiliated organizations, or those of the publisher, the editors and the reviewers. Any product that may be evaluated in this article, or claim that may be made by its manufacturer, is not guaranteed or endorsed by the publisher.
