# Supplementary material for: Evasion of toll-like receptor recognition by Escherichia coli is mediated via population level regulation of flagellin production
Source: Front Microbiol. 2023 Mar 23;14:1093922. doi: 10.3389/fmicb.2023.1093922 (PMC10078357; doi:10.3389/fmicb.2023.1093922)
Supplement: Supplementary file 1 [file Data_Sheet_1.PDF]

Supplementary Data for Tan *et al*

Table S1: Motile Strains and associated data used in this study

Lab Strain ID	Source*	Flagellin Serotype	Average swarm diameter (cm)	Flagellar abundance §		Doubling Time	Reference
				Avg Fla / cell	% Fla+		
2743	NCTC10418	H21	5.78	2.35	82	23.4 ± 7.3	(22)
3373	CFT073	H1	7.02	1.59	58	15.6 ± 3.2	(21)
3398	PYL	H1	3.59	1.46	7	19.3 ± 6.5	This study
3406	ABU	H18	3.06			15.3 ± 2.3	
3408	UTI	H1	5.71	1.66	25	15.3 ± 3.6	
3409	UTI	H5	1.87			16.6 ± 4.3	
3411	UTI	H6	7.78			26.9 ± 10	
3412	UTI	H18	1.49	1.36	27	18.7 ± 4.3	
3414	UTI	H27	7.39			16.1 ± 0.8	
3415	BAC	H5	3.04			15.6 ± 3.7	
3417	UTI	H1	2.05			17.7 ± 6.6	
3419	ABU	H6	3.56			15.2 ± 3.9	
3424	PYL	H18	4.98			14.1 ± 2.9	
3425	PYL	H1	4.51			21.6 ± 8.1	
3692	ABU	H4	3.07			13.9 ± 1.7	
3693	ABU	H6	2.67	1.24	8	14.2 ± 0.4	
3694	ABU	H18	5.41			22.1 ± 8	
3695	ABU	H5	3.96	1.41	10	19.1 ± 8.7	
3697	ABU	H18	2.44	1.60	6	14.5 ± 2	
3698	ABU	H5	2.36			14.7 ± 1.4	
3699	ABU	H4	0.77			15.8 ± 0.9	
3701	UTI	H27	2.80	1.68	52	21.5 ± 4.1	
3702	UTI	H4	6.97	1.52	24	16.8 ± 1.7	
3703	UTI	H1	1.00	1.71	10	17.8 ± 5.3	
3704	UTI	H6	2.10			16.7 ± 3	
3710	ABU	H4	2.85			15.1 ± 2.9	
4738	ABU	H5	1.58	1.67	50	21.2 ± 8.4	(8)
4739	ABU	H1	2.41	1.62	35	20.6 ± 5.7	
4740	ABU	H1	2.25	1.44	57	16.1 ± 2.8	
4741	ABU	H18	2.10	1.81	11	19.7 ± 10.4	
4742	ABU	H18	2.25	1.14	19	14.4 ± 2.3	
4743	ABU	H5	3.67	1.62	36	15.9 ± 0.3	
4744	ABU	H18	1.67	1.45	46	16.8 ± 3	
4745	ABU	H6	2.00	1.14	7	16 ± 3.4	

§ Data shown to indicate the strains used in Figure 5

*PYL: Pyelonephritis; ABU: asymptomatic bacteriuria; UTI: Acute cystitis; BAC: Bacteraemia

Table S2 Non-motile strains used in Figures 1 and 2D.

Lab Strain ID	Doubling Time	Source*
3399	14.7 ± 1.4	ABU
3400	25.3 ± 7.9	ABU
3401	21.2 ± 6.9	UTI
3402	19.9 ± 7.6	BAC
3403	19.8 ± 10.2	UTI
3407	18.9 ± 7.1	UTI
3410	20.2 ± 5.3	BAC
3413	16.4 ± 1.3	ABU
3416	18.9 ± 7.6	ABU
3418	18.4 ± 1.9	ABU
3420	20.5 ± 1.9	ABU
3422	18.5 ± 7.4	PYL
3696	21.3 ± 7	ABU
3701	19.6 ± 4.7	UTI
3706	23.4 ± 6.7	UTI
3707	17.5 ± 6	UTI

*See Table S1 descriptors

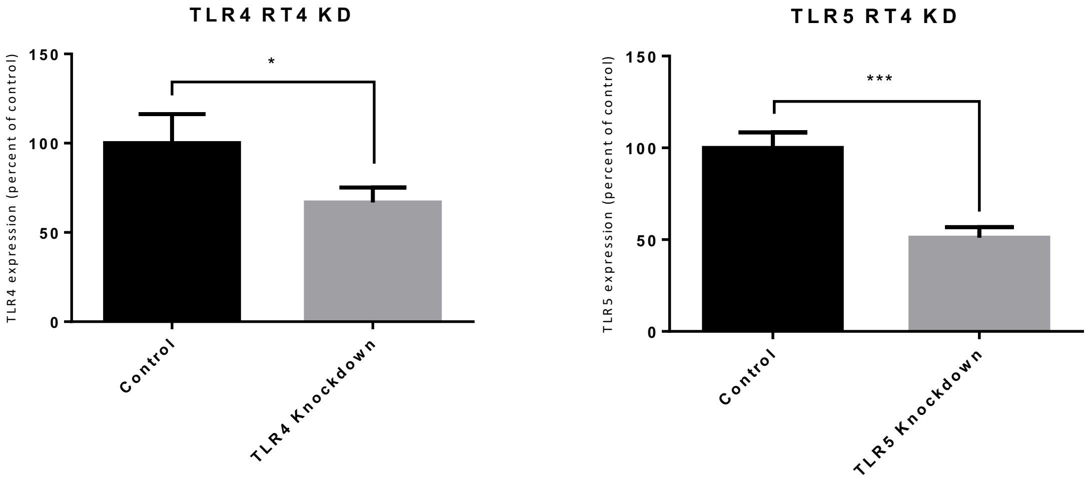

Figure S1: Quantification of expression in RT4 cells subjected to TLR4 and TLR5siRNA inhibition. Percentage knockdown efficiency of TLR4 and TLR5 via transfection with complementary siRNA.* indicated p<0.05, *** indicated p<0.0001. Graph titles define which gene was measured using qRT-PCR, see reference 30 for methodological details.

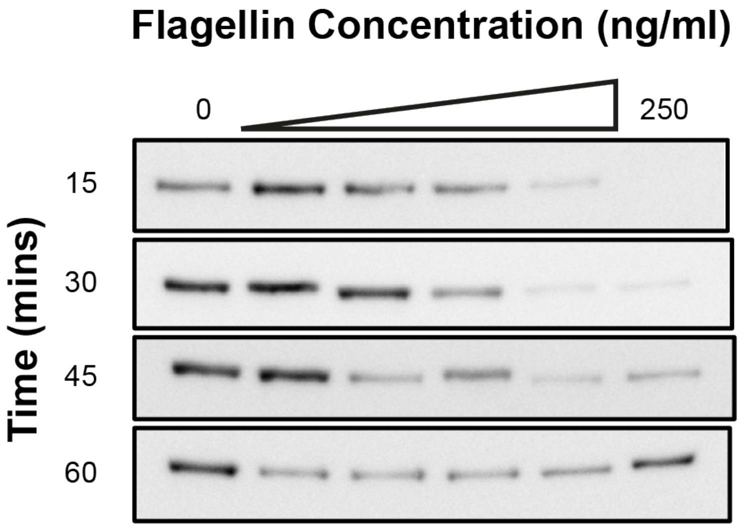

Figure S2: Analysis of Bladder RT4 cell NFκB induction via IκBα response following exposure to flagellar filaments. While the turnover and resynthesis of IκBα is rapid, the impact of NFκB activation is often measured in hours [Hayden and Ghosh (2004) Genes Dev. 18(18):2195–224]. Here we show that flagellin concentration is reflected by the turnover response that activates the NFκB signalling cascade. This correlates with the flagellar abundance data and the main conclusion of this work.

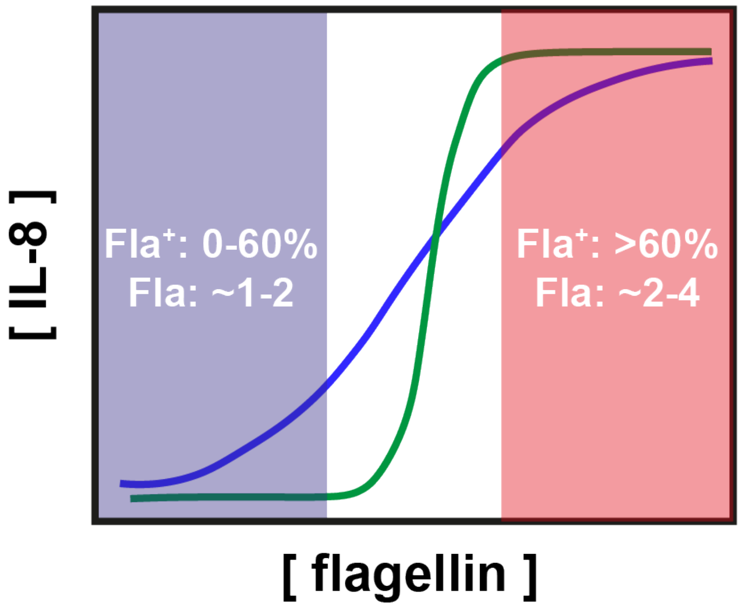

Figure S3: Working Model to highlight the conclusion of this study: that *E. coli* is able to adapt to maintain motility in a population but avoid TLR5 recognition. **Blue line**: The assumed linear correlation of flagellin concentration and IL-8 production based on *in vitro* analysis of TLR5 recognition. The data in the study argues recognition of *E. coli* will be dictated by host variability in TLR5 expression and the flagellated status of invading *E. coli* strains. This generates a window of recognition (**white area**) that to define its boundaries (**green line**) was outside the scope of this study. Understanding this interplay between host and microbe, however, is a research direction worth pursuing in order to appreciate the tolerance of *E. coli* invasion / colonisation of the urinary tract in UTI patients.
